# Cell-type-specific gene expression patterns in the knee cartilage in an osteoarthritis rat model

**DOI:** 10.1007/s10142-017-0576-6

**Published:** 2017-11-13

**Authors:** Michal Korostynski, Natalia Malek, Marcin Piechota, Katarzyna Starowicz

**Affiliations:** 1 Department of Molecular Neuropharmacology, Institute of Pharmacology Polish Academy of Sciences, Krakow, Poland; 2 Laboratory of Pain Pathophysiology, Department of Pain Pharmacology, Institute of Pharmacology Polish Academy of Sciences, Krakow, Poland; 3 Department of Neurochemistry, Institute of Pharmacology Polish Academy of Sciences, Smetna 12 Street, 31-343 Krakow, PL Poland

**Keywords:** Osteoarthritis, Gene expression, Time-course, Molecular profiles, Biomarkers

## Abstract

Osteoarthritis (OA) is a chronic degenerative disease that leads to joint failure, pain, and disability. Gene regulation is implicated as a driver of the imbalance between the expression of catabolic and anabolic factors that eventually leads to the degeneration of osteoarthritic cartilage. In our model, knee-joint OA was induced in male Wistar rats by intra-articular sodium monoiodoacetate (MIA) injections. Whole-genome microarrays were used to analyse the alterations in gene expression during the time-course of OA development (at 2, 14, and 28 days post-injection) in rat knee joints. The identified co-expressed groups of genes were analysed for enriched regulatory mechanisms, functional classes, and cell-type-specific expression. This analysis revealed 272 regulated transcripts (ANOVA FDR < 0.1% and fold > 2). Functionally, the five major gene expression patterns (A–E) were connected to PPAR signalling and adipogenesis (in cluster A), WNT signalling (in cluster B), endochondral ossification (in cluster C), matrix metalloproteinases and the ACE/RAGE pathway (in cluster D), and the Toll-like receptor, and IL-1 signalling pathways (in cluster E). Moreover, the dynamic profiles of these transcriptional changes were assigned to cellular compartments of the knee joint. Classifying the molecular processes associated with the development of cartilage degeneration provides novel insight into the OA disease process. Our study identified groups of co-regulated genes that share functional relationships and that may play an important role in the early and intermediate stages of OA.

**Electronic supplementary material:**

The online version of this article (10.1007/s10142-017-0576-6) contains supplementary material, which is available to authorized users.

## Introduction

Osteoarthritis (OA) is the most common type of arthritis and degenerative joint disease and involves cartilage and many of its surrounding tissues (Karsdal et al. 2016; Mobasheri and Henrotin 2015). Disease progression is usually slow but can ultimately lead to joint failure, pain, and disability. Hip and knee OA tend to cause the greatest burden to the population, as pain and stiffness in these large, weight-bearing joints often lead to significant disability, requiring surgical intervention (Litwic et al. 2013). OA is regarded as a complex disease whose cause is not completely understood. While it is perceived as a structural disease, the underlying pathology and chronic changes of OA occur at the cellular and molecular levels (Loeser 2006). Furthermore, effective diagnostic aids are not available to assist in the management of OA. Persons with OA are a heterogeneous population, ranging widely in age, disease impairment, functional goals, and interests (Nguyen et al. 2011). Therefore, the management of OA patients should be comprehensive and individualised and should take into account the progression rate of the disease. The genetics of OA are important for understanding its initiation and progression (Sandell 2012; Thakur et al. 2013). There is still a need for molecular markers in OA for disease diagnosis, prognosis, and monitoring in clinical practice, as well as for patient selection and study design optimization in clinical interventional trials (Karsdal et al. 2016).

Understanding the molecular events that occur within articular cartilage will provide knowledge not only of the disease mechanisms but also of new diagnostic markers and cellular targets for therapeutic and nutritional interventions. Although clinically subtypes can be defined by their aetiology, clinical presentation, and radiographic evaluation, how these subtypes translate into the cellular and molecular pathways of joint degradation remains unknown. Insights into osteoarthritis progression have revealed that different cell-type populations might be important for disease progression. Several studies have suggested a role for synovial macrophages and their main proinflammatory cytokines [interleukin (IL)-1 tumour necrosis factor (TNF)-α] in driving OA synovitis (Bondeson et al. 2006). However, the complex aetiology of OA indicates the involvement of diverse roles and multiple cell types in the process of cartilage degeneration.

While exciting breakthrough treatments continue to become available for rheumatoid arthritis (RA), highly effective therapies do not exist for OA. With a better understanding of the molecular process through continued research, progress may be achieved in the development of medications to effectively control the symptoms and disease progression of OA. The imbalance between anabolic and catabolic factors that leads to the breakdown and degradation of articular cartilage in OA involves many factors: proteins (including structural proteins of the extracellular matrix) inflammatory cytokines, catabolic and anabolic enzymes, and cell-signalling molecules (Rousseau and Delmas 2007).

A functional genomic approach to OA focuses on measuring changes in gene expression, allowing for the discovery of new factors involved in the disease and in joint tissue development or maintenance (Steinberg et al. 2017; Steinberg and Zeggini 2016). Further understanding of the molecular and cellular basis of OA is fundamental for the identification of new therapeutic targets and the development of specific strategies and interventions (Alcaraz et al. 2010). We used a transcriptomics study to compare data between healthy and various OA states to better understand the mechanisms underlying the disease and to guide the development of new therapeutic strategies.

## Methods

### Animals

Male Wistar rats (Charles River, Germany) initially weighing between 225 and 250 g were used for all the experiments. Animals were housed 5 per cage under a standard 12/12 h light/dark cycle and had free access to food and water. The animal procedures were performed following the recommendations of the International Association of Studies on Pain and the reduce, replace, and refine principles (3Rs). The study was approved by the Local Bioethics Committee of the Institute of Pharmacology PAS (approval number 938/2012). In the experiments, we compared non-treated (intact) and osteoarthritic animals. Our previous results showed no significant changes in joint hypersensitivity and DWB tests for saline-injected rats (unpublished data). Since our study aimed at the comprehensive comparison between healthy and OA-affected cartilage in the context of existing pain, this approach, respecting the 3R principles, seems to be perfectly sufficient. Rats were randomly allocated to the groups.

### MIA-induced model of osteoarthritis

The rats were anaesthetized with 5% isoflurane (Forane, Baxter Healthcare Corporation, USA) in 100% O_2_ (3 L/min) until the flexor withdrawal reflex was abolished. The skin covering the right knee joint was shaved and swabbed with 100% ethanol. A 27-gauge needle was introduced into the joint cavity through the patellar ligament, and 50 μl containing 3 mg of sodium monoiodoacetate (MIA; Sigma-Aldrich, USA) in 0.9% saline was injected intra-articularly (i.a.) to induce OA-like lesions. The MIA model was chosen due to its particular usefulness for studying joint pain and due to multiple similarities in the mechanisms of cartilage degeneration in this model to those observed in human OA cartilage. The dose of MIA (3 mg i.a., injection) was selected based on previous investigations in rats (Cao et al. 2017; Nguyen et al. 2011; Tan et al. 2006).

### Tissue collection and RNA isolation

**Fig. 1:**
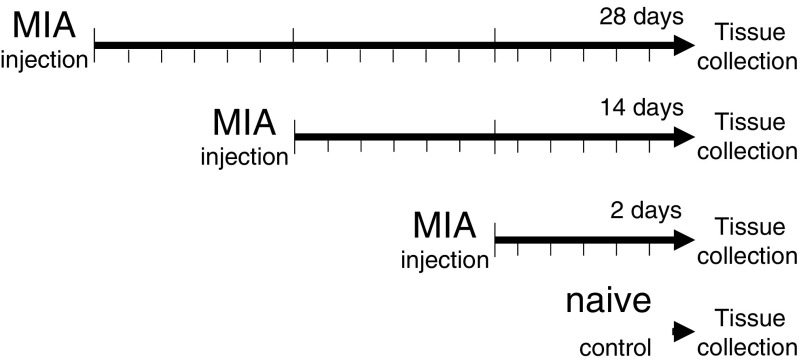
Outline of the experimental design. The rat model of OA was developed by the intra-articular (i.a.) injection of 3 mg of MIA. Rats were monitored for OA-related pain symptoms before MIA injection and every second day after the procedure. The animals were sacrificed at days 2, 14, and 28 post-MIA injection. Joint tissue samples (consisting of the meniscus and articular cartilage from the tibia and femur) from the ipsilateral knees were collected from 32 animals (*n* = 8). Whole genome expression profiling using microarrays was performed at the selected time-points

The animals were sacrificed at days 2, 14, and 28 post-MIA injection (Fig. 1). The time-dependent alterations that occur following MIA induction mimic the changes observed in human pathology. In our previous work, we monitored the development of pain-like behaviour in rats during the 28 days of MIA-induced OA progression (Pajak et al. 2017). The ipsilateral knee was opened with surgical instruments, and the meniscus and the articular cartilage of the tibia and femur were collected. Because of mechanical nature of the isolation, the samples include small parts of joint capsule and bone. The collected samples were placed in individual tubes containing the tissue storage reagent RNAlater (Qiagen Inc., Valencia, CA, USA) and stored at − 70 °C until RNA isolation. Each sample consisted of the ipsilateral knee tissue from one individual animal, and 4 replicate samples were analysed per group, i.e., the analysis is based on 8 rats per group. RNA was isolated following the manufacturer’s protocol and further purified using an RNeasy Mini Kit (Qiagen Inc.). The total RNA concentration was measured using an ND-1000 Spectrometer (NanoDrop Technologies Inc., Montchanin, DE, USA).

### Microarray analysis

The quality of RNA was determined by using an RNA 6000 Nano LabChip Kit and Agilent Bioanalyser 2100 (Agilent, Palo Alto, CA, USA). Preparation of cRNA was performed according to the protocol provided by Affymetrix (Santa Clara, CA). The same amounts of total RNA from two animals were pooled and further purified by using an RNeasy Mini Kit (Qiagen Inc. Total RNA (3 μg) derived from each pool (*n* = 4) was converted to double-stranded cDNA using a SuperScript System (Invitrogen, Carlsbad, CA) and an oligo(dT)24 primer containing a T7 RNA polymerase promoter site. Biotin-labelled cRNA was synthesised from cDNA using a labelling Kit and purified by using a GeneChip Cleanup Sample Module (Qiagen Inc., Valencia, CA, USA). The yield of the in vitro transcription reaction was determined by the product absorbance at 260 nm, as measured by NanoDrop ND-1000 (NanoDrop Technologies, Inc., Montchanin, DE), size of cRNA probes was evaluated by using RNA 6000 Nano LabChip Kit (Agilent, Palo Alto, CA, USA). Fragmented cRNA was used for hybridization to GeneChip® Rat Gene 2.0 ST arrays (Affymetrix). The arrays were washed and stained with streptavidin-phycoerythrin (Merck, Darmstadt, Germany) in Fluidics Station 400 (Affymetrix) according to the standard protocol of the manufacturer. The arrays were scanned by using a GeneChip Scanner 3000 (Affymetrix).

### Microarray data analysis

Microarray data were initially processed using GeneChip Operating Software (Affymetrix) with the appropriate quality controls. After background subtraction, the data were normalised using quantile normalisation and then log_2_-transformed. The obtained signal was considered the measure of mRNA abundance, derived from the level of gene expression. All statistical analyses were performed in the latest version of R software. Statistical analysis of the results was performed using appropriate statistical tests, followed by corrections for multiple testing (estimation of false discovery rate). Hierarchical clustering was performed using the measure of Euclidian distance and the average distance linkage methods. Cluster visualisation was performed using dChip software (www.dchip.org).

### Functional classification and cell-type-specific gene expression

The gene annotation tool Enrichr was used to identify over-represented ontological groups among the gene expression patterns and to group the genes into functional classifications (Kuleshov et al. 2016). Over-represented terms (Wiki Pathways 2016) were defined as having at least three transcripts and *p* < 0.05 per Fisher’s exact test. For cell-type enrichment of mRNA, the Enrichr cell types (Gene Atlas) module was used. The identification of over-represented transcription factor binding sites (TFBSs) in the regulatory regions of genes was performed using the Seqinspector resource (Piechota et al. 2016). Gene symbol lists were submitted, and default parameters were used.

## Results

### Gene expression profiling during the development of knee-joint arthritis

We used whole-genome Affymetrix Rat Gene 2.0 ST microarrays to analyse the alterations in gene expression overtime in rat knee joints following MIA treatment. The early, intermediate, and relatively late changes in mRNA abundance were analysed at three time points (2, 14, and 28 days following MIA injection) and compared to the mRNA expression of healthy animals (Fig. 1). Microarray data analysis using one-way ANOVA identified 884 transcripts (expected number of false positives ~ 0) with altered expression levels during the development of knee-joint arthritis. Further data analysis indicated 272 transcripts that had significantly different expression levels between the intact control and MIA-treated animals (*t* test; *p* < 0.0001 corresponding to FDR < 0.1%) for at least one of the time points (fold change > 2). The false discovery rate was estimated using permutation analysis (1000 permutations), which was implemented by dChip software. The list of 241 annotated genes (from the list of 272 transcripts) was used in the following analyses. A table listing the gene expression profiling results of MIA treatment in the knee joint is provided in Supplementary Table S1.

### Dissection of gene expression patterns

**Fig. 2:**
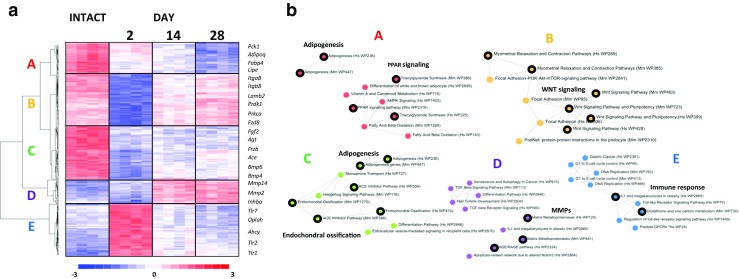
Profiling of gene expression alterations in the rat model of knee-joint arthritis. **a** Hierarchical clustering of MIA-induced transcriptional alterations in knee cartilage. Microarray results are shown as a heat map and include genes that had genome-wide significance during at least one of the time points in the comparison of intact vs. MIA-treated groups. Coloured rectangles represent transcript abundance 2, 14, and 28 days after the injection of MIA, as indicated above. The intensity of the colour is proportional to the standardised values (between − 3 and 3) from each microarray, as indicated on the bar below the heat map image. Hierarchical clustering was performed with the dChip software using Euclidean distance and the average linkage method. Major MIA-induced gene transcription patterns are arbitrarily designated as clusters A–E. Example genes from clusters A–E are labelled on the right. The presented genes are involved in the functional pathways enriched for that particular cluster (Supplementary Table S3). **b** The graphs indicate the functional associations between the genes in clusters A–E that are regulated in response to MIA treatment. Functional links were identified using Wiki Pathways 2016 and visualised by the Enrichr online application. The top three functional pathways (sorted by *p* < 0.05) are indicated

**Fig. 3:**
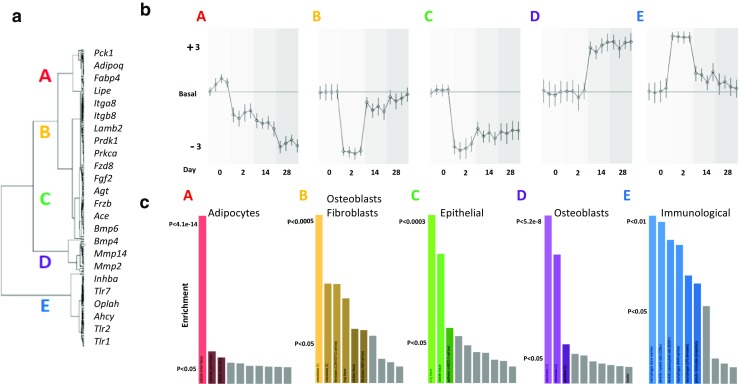
The enrichment in the expression patterns of OA-regulated genes in various types of cells and tissues. **a** Gene clustering presents the co-expressed patterns A–E (please see Fig. 2) with the example genes labelled on the right. **b** The average activity of time-dependent MIA-induced gene expression patterns. The results are presented as the mean change in gene expression (measured using z values in the A–E groups of genes). The values are relative to the level of transcript abundance in naïve animals (at each of the time points, i.e., 2, 14, and 28 days). **c** The top ten enriched cell/tissue types are presented in the graphs (corrected and nominal *p* values are presented using the colours). The emerging types are indicated. The enrichment in the expression of MIA-regulated gene clusters in tissues and cell types from the Mouse Gene Atlas was analysed using Enrichr

Hierarchical clustering revealed five major gene transcription patterns associated with the development of knee-joint arthritis in a rat model of osteoarthritis (arbitrarily described as A–E; Fig. 2). Five main MIA-responsive gene clusters, representing patterns A (containing 34 transcripts), B (56 transcripts), C (59 transcripts), D (29 transcripts), and E (63 transcripts), became evident. The clusters revealed diverse time-dependent patterns of up- and downregulated gene expression levels (Fig. 3b). Pattern A consisted of genes displaying a continuous decrease in their expression levels during days 2–28 of arthritis development. The expression levels of genes in pattern B were substantially downregulated 2 days after MIA treatment and returned to close to basal level after 28 days. Pattern C contained genes that were rapidly downregulated after treatment, and this effect was maintained until day 28. Two gene clusters were upregulated; in one cluster, this pattern was exhibited during days 14–28 (pattern D), and in the other cluster, it occurred at day 2 (pattern E) after the MIA injection.

### Identification of regulatory factors

To investigate the mechanisms of MIA-induced alterations in gene transcription, we searched for over-represented transcription factor binding sites in the regulatory regions of co-expressed transcripts (Supplementary Table S2). We exploited available ChIP-seq data (Consortium 2012) using the newly developed, online resource Seqinspector (Piechota et al. 2016). Promoters of downregulated transcripts (pattern A) exhibited a significant overrepresentation in the ChIP-seq signal for the PPARG (peroxisome proliferator-activated receptor) transcription factor (*p* = 1.6E^−6^, *t* test with Bonferroni correction). Transcripts activated relatively early in the response to MIA treatment (pattern E) represented a set of transcriptional regulators, including STAT3 (*p* = 1.1 × 10^−7^), CEBPB (*p* = 1.4 × 10^−7^), IRF4 (*p* = 3.5 × 10^−7^), RELA (*p* = 3.7 × 10^−7^), and RELB (*p* = 4.6 × 10^−7^). Surprisingly, despite a robust co-expression of genes with transcriptional patterns in clusters B, C, and D, the identification of regulatory factors was difficult. We found a slightly higher binding of the muscle-specific transcription factor MYOG on promoter regions of genes in the cluster B expression pattern (nominal *p* = 0.06).

### Functional classification of MIA-regulated genes

To identify functional associations between genes with similar expression profiles induced by MIA, we used the Enrichr gene list enrichment analysis tool. A list of genes from each gene expression pattern was analysed by the Wiki Pathways 2015 categories (Fig. 2b). The group of genes in pattern A was enriched with factors involved in PPAR signalling (e.g., *Adipoq* and *Aqp7*) and adipogenesis (e.g., *Cfd* and *Lipe*). Among the genes in pattern B, transcripts connected with WNT signalling (e.g., *Fzd8* and *Prkca*) and focal adhesion (e.g., *Itga8* and *Itgb8*) were over-represented. Genes involved in endochondral ossification (e.g., *Frzb* and *Fgf2*) and semaphoring interactions (e.g., *Myh11* and *Sema3e*) were over-represented within the C expression pattern. According to the transcript expression level, the enriched genes in cluster D were connected to matrix metalloproteinases and the ACE/RAGE pathway (e.g., *Mmp14* and *Mmp2*), whereas analysis of cluster E revealed the enrichment of genes involved in Toll-like receptor and IL-1 signalling pathways (e.g., *Trl1*, *Trl2*, and *Il18*).

### Cell-type specific expression of MIA-regulated genes

We further investigated whether the alterations in gene expression were associated with particular cell types (Fig. 3c). Identification of the types of cells expressing genes in clusters A–E was carried out in reference to the Mouse Gene Atlas, which catalogues the cellular enrichment of individual transcripts in various cells and tissues. A significant enrichment of transcripts from the gene pattern A are found in adipose brown (e.g., *Cfd* and *Dgat2*). Gene clusters B (e.g., *Timp2* and *Fzd8*) and D (e.g., *Col8a1* and *Lbp*) are enriched with genes that are expressed in osteoblasts. In addition, cluster D also contains genes that are expressed in neuronal cells. Enriched transcripts from pattern C are found in the lung, bladder and mouse embryonic fibroblasts (e.g., *Galntl4* and *Tsn1*). Gene network E was characterised by a strong overrepresentation of genes that are expressed in dendritic plasmacytoid B220+ (e.g., *Irf8* and *Tlr7*) and multiple types of macrophages. A detailed description of the results from the functional classification and cell-type enrichment is included in Supplementary Table S3.

## Discussion

Profiling gene expression alterations over time represents a promising approach for understanding OA aetiology (Korostynski et al. 2017; Nam et al. 2011; Wang et al. 2015). In past years, several studies that aimed to determine the involvement of gene regulation in the development of cartilage degeneration have been published (Li et al. 2014; Ramos et al. 2014). These studies focused on knee and hip cartilage and provided transcriptional markers of disease progression (Wang et al. 2016). However, the dynamic changes in cartilage structure and function are associated with a variety of cell-type-related biological processes that are simultaneously activated and inhibited (Dunn et al. 2016). Therefore, the approaches that aim to investigate patterns of molecular alterations across cellular compartments in diseased tissue might provide novel insight into the mechanisms of disease development (Aigner et al. 2006).

In the present study, we used whole-genome microarrays to define the sequence of molecular changes associated with the development of OA pathology. A total of 241 differentially expressed genes were identified between OA and healthy cartilage. Our analysis revealed that the transcriptional response to the MIA injection was divided into five major groups of co-regulated genes. The identified groups were similar in size (between 30 and 60 transcripts) and exhibited specific time-dependent alteration profiles: three clusters had downregulated gene expression (A, B, C), while two clusters were upregulated (D, E). Exploring these dynamic changes indicated a functional connection between transcripts with similar regulation patterns (up- or downregulated) of mRNA abundance levels (Loeser et al. 2012).

The first identified pattern (pattern A) consisted of gradually downregulated genes, which have an enriched expression in adipose tissue at basal conditions. These genes are functionally connected to the PPAR signalling pathway and adipogenesis. This is supported by the known functions of these particular genes from this cluster, including involvement in gluconeogenesis (*Pck1*), glucose regulation (*Adipoq*), energy storage mobilisation (*Lipe*), and fatty acid metabolism (*Fabp4*). Moreover, using bioinformatic analyses, we were able to determine the role of the PPARG (PPAR-gamma) transcription factor as an important regulator of the genes in this pattern (Fahmi et al. 2011). PPARG is a key regulator of cartilage health and ablation of this gene in mice leads to an accelerated OA phenotype (Vasheghani et al. 2015). A decrease in PPARG activity in OA would therefore reflect a decrease in the anti-inflammatory potential of this disease (Kobayashi et al. 2005). Some evidence indicates that the infrapatellar fat pads in osteoarthritic joint tissues are capable of modulating inflammatory and destructive responses in knee OA (Clockaerts et al. 2010; Mariman and Wang 2010). The mechanism connecting the progressive reduction in the expression of the identified group of transcripts with the development of MIA-induced OA is unknown. However, it might be speculated that anti-inflammatory mediators secreted by adipose tissue into synovial compartments are important for the suppression of inflammation and chronic pain. Interestingly, the ageing-associated decrease in IL-1β-stimulated leptin production has been suggested as protective against the development of OA in F344BN rats (Fu et al. 2016). There is evidence that the activation of PPARG2 by the antidiabetic drug rosiglitazone stimulates adipogenesis and inhibits osteoblastogenesis in different mesenchymal progenitor models in vitro and in vivo (Rzonca et al. 2004). Therefore, it has been suggested that an early event in the initiation and progression of OA is a preferential shift towards osteoblastogenesis that results from the downregulation of PPAR signalling (Tan et al. 2006; Watters et al. 2007). The transdifferentiation of adipocytes into osteoblasts has been suggested as a process involved in the pathogenesis of OA (Lecka-Czernik et al. 2002). It may provide important insight into the impaired removal of old or damaged bone by osteoclasts and the subsequent replacement with new bone formed by osteoblasts.

The group of co-regulated transcripts in cluster B was enriched with genes expressed predominantly in osteoblasts. Genes from this group encode compounds of the integrin complex (*Itga8* and *Itgb8*) and lamins (*Lamb2*), which mediate interactions between the cell and extracellular matrix. Cluster B was also enriched with genes involved in the WNT signalling pathway (including *Prkca* and *Prkd1*) and in the control of muscle contraction and relaxation (*Igfbp6* and *Atf5*). There is accumulating evidence that WNT signalling plays a critical role in the repression of osteo-chondrodifferentiation and the synthesis and turnover of the cartilage matrix (Luyten et al. 2009). The identified group of genes displayed a rapid decrease in expression levels 2 days after MIA-induced OA and increased back to control level at day 28. The observed molecular effects might be associated with the process of osteo-chondrogenesis and recovery of cartilage functions (Cao et al. 2017; Goldring 2012).

The third gene expression pattern, cluster C, was rapidly downregulated in response to MIA treatment. The effect was permanent with only a slight increase in the mean mRNA expression level during the experiment. Our bioinformatics approach indicated that the genes in this group are functionally connected with endochondral ossification. Our results also indicated that these transcripts are predominantly expressed in epithelial tissues (such as lung or bladder tissues). This group included genes in the ACE angiotensin-converting pathway (*Ace* and *Agt*) and of bone morphogenetic proteins (*Bmp6* and *Bmp4*). The connection between ACE polymorphisms and osteoarthritis susceptibility has been identified in genetic association studies in humans (Qing and Ye 2015). It is possible that a decrease in the mRNA expression level of genes involved in the ACE pathway may accelerate cartilage degeneration. Notably, there are clinical reports suggesting that treatment with ACE inhibitors may influence the development of arthritis disorders (Verdecchia et al. 2010).

Two of the identified gene clusters showed an increase in expression level during the time course of the experiment. Pattern D contained genes significantly enriched in osteoblasts. These transcripts showed a relative upregulation in expression levels between days 14 and 28 after MIA-induced knee OA. The profile showed no differences in gene expression levels at day 2 compared to those of the controls animals. In this group, we found an overrepresentation of genes connected to matrix metalloproteinases (MMPs). MMPs are key enzymes in the turnover of the extracellular matrix that play an important role in resistance to compressive forces and in maintaining the tensile properties of tissue. It is well known that MMP gene expression is upregulated in OA and is connected to the degradation of major extracellular matrix components (Yoshihara et al. 2000). Experimental interventions to restore the imbalance between anabolism and catabolism include MMP subtypes (Yang et al. 2013). Our gene expression profiling indicated that MMP14 and MMP2 are potential pharmacological targets in OA treatment. The enduring increase in MMP expression confirms that these enzymes are important factors in the development and maintenance of OA (Pajak et al. 2017; Yang et al. 2013).

The last pattern, cluster E, expressed genes involved in the immunological response. The profile indicated a rapid induction of these genes after MIA injection, but the expression levels returned to basal by day 28. Recent research has indicated that OA pathogenesis is driven by an immune response that progressively catalyses degenerative changes, ultimately leading to an altered joint microenvironment (Kandahari et al. 2015). We have confirmed the early transcriptional activation of genes involved in IL-1 and Toll-like receptor signalling pathways. The response was enriched by genes expressed in macrophages and dendritic cells. As OA-consistent changes in the joint occur rapidly following injury and are associated with inflammation, anti-inflammatory pharmacological approaches should be aimed at the early reactive phase of OA pathogenesis. Gene expression profiling results agree with clinical observations that the use of anti-inflammatory drugs may be less effective at later stages of OA (Yang et al. 2013). In addition to the traditional inflammatory cytokines such as IL-1β, IL-6, and TNF-α, damage-associated molecular patterns have been implicated as drivers of the chronic, low-level inflammation associated with OA (Liu-Bryan and Terkeltaub 2015). These molecules can activate pattern recognition receptors (PRRs) on chondrocytes and synovial macrophages and thus promote cartilage degradation and synovitis in OA (Liu-Bryan 2013). These PRRs include TLR; TLR4, in particular, has been highlighted as a potential target for disease-modifying OA drugs (Malek et al. 2015; Miller et al. 2015).

OA pathogenesis is multifactorial and complex, as evidenced by unique phenotypes and seemingly discrete stages (i.e., early, intermediate, and late) (Heinegard and Saxne 2011). We have monitored transcriptional alterations in an animal model that reproduces the time-course of OA development. The identified groups of co-regulated genes present various insights into the molecular processes occurring in OA-affected knee cartilage. The coordinated decrease and increase in mRNA expression levels of functionally connected factors indicates numerous biological pathways. The most distinct molecular processes are related to PPARG- and WNT-signalling and the activation of MMP expression and the inflammatory response (Vasheghani et al. 2015). Moreover, these patterns revealed significant differences in the expression of transcripts enriched in specific types of cells and tissues. Based on these results, we assigned the identified molecular pathways to cellular compartments of OA-affected knee cartilage. The processes may have different or opposite roles in the regulation of catabolic and anabolic processes, but all the alterations eventually lead to joint degeneration.

Several potential limitations should be acknowledged. First, our analysis of cell-type-associated gene expression relied on a database of basal transcriptional profiles from various tissues and cell types rather than the gene expression profiles of OA-affected knee cartilage. We have included the most comprehensive dataset of gene expression profiling available in various cell types, and the obtained results are scientifically sound. Additional single-cell, transcriptomic studies are required to better understand the complex profile of gene expression alterations associated with the process of OA development. Second, the limitations of our strategy include potential differences in OA aetiology between the human and animal model, and the extreme complexity of the analysed tissue. Third, this study was limited to transcriptional mechanisms activated in response to MIA administration. We previously validated the use of the MIA-induced model of knee OA in rats (Malek et al. 2015). Despite these limitations, the obtained results may provide new insights into the molecular control of the early and intermediate phases of OA development.

## Conclusions

Our study provides evidence that the progression of cartilage damage is driven by the complex but precise regulation of gene patterns, which are induced or suppressed during various stages of cartilage damage. We see a dramatic, temporary loss of transcripts involved in the WNT signalling pathway and a constant downregulation of genes related to endochondral ossification. However, the decrease in the transcript abundance of genes connected to PPARG-signalling was more gradual. Transcripts related to the immunological response showed an early spike in expression levels, while genes related to matrix metalloproteinases exhibited a delayed increase in abundance levels. We conclude that the expression of PPARG-signalling genes correlates negatively with the different stages of OA and that the expression of matrix metalloproteinase genes correlates positively with OA development. Moreover, our results suggest that the observed transcriptional alterations are located in diverse cellular compartments of knee cartilage. We can speculate that synoviocytes, osteoblasts, epithelial fibroblasts, and immunological cells process specific, complementary, or oppositional physiological programmes during OA pathogenesis. The presented classification of transcript alterations that are associated with the development of cartilage degeneration provides novel insight into the OA disease process.

## Electronic supplementary material

Supplementary Table S1 A table listing the results from the gene expression profiling of the MIA-treatment effects in the knee joint. mRNA abundance levels of 272 genes were found to be significantly different between the intact control and MIA-treated animals (*t* test; *p* < 0.0001 corresponding to FDR < 0.1%) during at least one of the time points (fold of change > 2). The second sheet contains lists of the genes from the five major gene expression networks (A–E). (XLSX 49 kb)

Supplementary Table S2 The results of the TFBS analysis of the regulatory regions of the MIA-regulated genes. The table contains lists of the genes with experimentally validated binding sites for particular regulatory factors (with the average coverage of the ChIP-seq signal at > 1). Only transcription factors with a significant overrepresentation in the indicated gene cluster were included in the table. The analysis was performed using the Seqinspector resource available at http://seqinspector.cremag.org (Piechota et al. 2016). (XLSX 16 kb)

Supplementary Table S3 The results of the functional enrichment analyses performed with the Enrichr tool for the five clusters of genes significantly altered by the MIA injection. The table consists of an enriched term, the number of input genes in the pathway (overlap), the *p* value (*p* < 0.05), the z score, the combined score (computed as logarithm from *p* value from the Fisher’s exact test multiplied by the z score of the deviation from the expected rank), and the names of overrepresented genes. (XLSX 25 kb)

## Abbreviations

OA: osteoarthritis
MIA: monoiodoacetate
PPR: pattern recognition receptors
